# Sustainable Soil Additives for Water and Micronutrient Supply: Swelling and Chelating Properties of Polyaspartic Acid Hydrogels Utilizing Newly Developed Crosslinkers

**DOI:** 10.3390/gels10030170

**Published:** 2024-02-27

**Authors:** Youssef Hafidi, Hicham El Hatka, Dominik Schmitz, Manuel Krauss, Jürgen Pettrak, Markus Biel, Najim Ittobane

**Affiliations:** 1 Molecular Chemistry and Organic Materials Team (CMMO), Faculty of Science, Moulay Ismail University, Meknes 50050, Morocco; youssef.hafidi@edu.umi.ac.ma (Y.H.); hicham.elhatka@edu.umi.ac.ma (H.E.H.); n.ittobane@umi.ac.ma (N.I.); 2 IAP—Institute for Applied Polymer Chemistry, University of Applied Sciences Aachen, 52428 Jülich, Germany; dominik.schmitz@fh-aachen.de (D.S.); pettrak@fh-aachen.de (J.P.); 3 Research Institute of Water Management and Climate Future at RWTH Aachen University e.V., 52072 Aachen, Germany; krauss@fiw.rwth-aachen.de

**Keywords:** polyaspartic acid, glycine, superabsorbent polymers, hydrogels, biodegradable polymers, swelling properties, micronutrients

## Abstract

Drought and water shortage are serious problems in many arid and semi-arid regions. This problem is getting worse and even continues in temperate climatic regions due to climate change. To address this problem, the use of biodegradable hydrogels is increasingly important for the application as water-retaining additives in soil. Furthermore, efficient (micro-)nutrient supply can be provided by the use of tailored hydrogels. Biodegradable polyaspartic acid (PASP) hydrogels with different available (1,6-hexamethylene diamine (HMD) and L-lysine (LYS)) and newly developed crosslinkers based on diesters of glycine (GLY) and (di-)ethylene glycol (DEG and EG, respectively) were synthesized and characterized using Fourier transform infrared (FTIR) spectroscopy and scanning electron microscopy (SEM) and regarding their swelling properties (kinetic, absorbency under load (AUL)) as well as biodegradability of PASP hydrogel. Copper (II) and zinc (II), respectively, were loaded as micronutrients in two different approaches: in situ with crosslinking and subsequent loading of prepared hydrogels. The results showed successful syntheses of di-glycine-ester-based crosslinkers. Hydrogels with good water-absorbing properties were formed. Moreover, the developed crosslinking agents in combination with the specific reaction conditions resulted in higher water absorbency with increased crosslinker content used in synthesis (10% vs. 20%). The prepared hydrogels are candidates for water-storing soil additives due to the biodegradability of PASP, which is shown in an exemple. The incorporation of Cu(II) and Zn(II) ions can provide these micronutrients for plant growth.

## 1. Introduction

Water scarcity and drought conditions have become major challenges, contributing to desertification and threatening global agricultural productivity and food security [1,2,3,4]. In response to these problems, it is becoming imperative to advance research into improving water use efficiency in the agricultural sector. Therefore, the development of innovative materials to enhance water management and retention in soils has received increased attention. Among these materials, superabsorbent polymers (SAPs) are of interest. They are crosslinked hydrophilic polymers, which are able to absorb and retain large quantities of water or aqueous media [5].

Their application in soil can increases its water-holding capacity as well as the time between irrigation events and leads to a decrease in drought stress for the plants [6]. However, the majority of available products are based on polyacrylic acid (PAA), which has limited biodegradability and is based on fossil resources [7]. Even though PAA-based hydrogels are helpful materials with good performance under controlled conditions (especially for use in combination with deficit irrigation [8]) more work on the development of hydrogels for agricultural applications is needed to create future products with a strong focus on sustainability aspects, functionality, and performance.

Besides polysaccharide-based hydrogels, polyamino acids are promising candidates to form biodegradable sustainable soil amendments. Crosslinked poly(aspartic acid) (PASP) has proven to be a good alternative to PAA. It is a biodegradable polymer containing large amounts of carboxylate groups in its neutralized form. Due to these structural similarities to PAA, it takes up large quantities of water, too, if crosslinked [9,10,11]. Furthermore, the monomeric unit aspartic acid (ASP) can be produced from renewable resources and residuals via the catalytic conversion or fermentation of biomass. Nevertheless, more research is needed in this field [12,13].

The synthesis of PASP-based hydrogel is conducted via the thermal polycondensation of aspartic acid under acidic conditions to prepare polysuccinimide (PSI) with a high molecular weight [14]. PSI as an intermediate compound can easily be modified by amines [15]. Therefore, crosslinking is usually carried out in a solution, e.g., in N,N′-dimethylformamide (DMF), upon the addition of a suitable diamine crosslinker. Basic hydrolysis with, e.g., NaOH, results in the PASP hydrogel in its sodium salt form. The specific reaction conditions, such as the temperature, crosslinking time, concentration of the polymer, molecular weight of PSI, concentration of the crosslinking agent, hydrolysis conditions, and drying conditions, have an influence on the properties and swelling ratio of the resulting PASP hydrogels [16].

Diamines are commonly used as crosslinking agents for PSI. Yang et al., used 1,6-hexamethylene diamine (HMD) to form PASP hydrogels and determined the degradability in an α-chymotrypsin solution [17]. 1,4-diaminobutane was used by Krisch et al., to prepare PASP-hydro- and nanogels [18]. In addition, primary amines derived from natural products can open succinimide rings. Amino acids and derivatives, such as lysine-methylester and cystamine (CYS), can be used as crosslinkers for the synthesis of PASP hydrogels. Lysine methylester partially decreases the anionic character of aspartic acid. CYS is biologically active and has the ability to provide free thiol groups under reductive conditions [19].

In addition to the function as a water reservoir, hydrogels can contribute to the nutrient supply of plants. A carboxymethyl cellulose (CMC)-based nanocomposite hydrogel was studied for the encapsulation of an NPK fertilizer [20]. Besides macronutrients such as NPK, micronutrients are important for plant growth and health. Among others, copper and zinc are two important micronutrients. Copper is present in several cellular processes in plants, e.g., among other functions, it is essential for photosynthesis or carbon and nitrogen metabolism. Zinc plays an important role as a component in enzymes for the synthesis of proteins to name one example [21,22]. It is also important for the plants’ immune system [23]. The encapsulation of such micronutrients in hydrogels was studied by Ekanayake et al., who synthesized an alginate-based hydrogel loaded with ZnO and CuO nanoparticles [24]. Copper-loaded alginate-CMC-based hydrogels were prepared by Skrzypczak et al. [25]. Zinc-loaded CMC- and carrageenan-based hydrogels provided positive effects on the growth of wheatgrass [26].

**Scheme 1:**
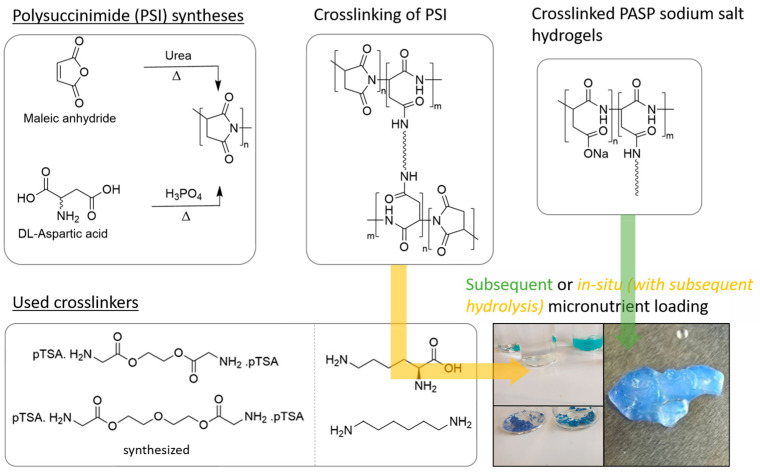
General overview of the study carried out in this work.

In this study, newly developed diamine crosslinkers made of glycine (GLY) attached to diol components were synthesized and used among other diamines as crosslinkers for PSI. In addition, copper and zinc salts were used exemplarily for the incorporation of micronutrients (Scheme 1). Overall, a toolbox for sustainable soil additives was developed, which can easily be modified to fulfill specific needs like water uptake, uptake speed, absorbency under load, the incorporation of micronutrients, and biodegradability.

## 2. Results and Discussion

### 2.1. Syntheses of Glycine Crosslinkers and PSI

#### 2.1.1. Crosslinker Syntheses

**Scheme 2:**
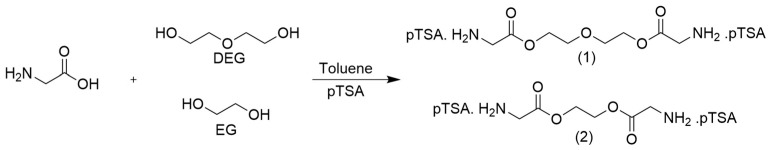
Reaction between glycine and (1) DEG and (2) EG.

Besides the diamines HMD and LYS, bifunctional glycine derivatives of EG and DEG were successfully synthesized by classical-acid-catalyzed, azeotropic esterification using *p*-toluenesulfonic acid as a catalyst, resulting in the compounds di-*p*-toluenesulfonic acid bis-(glycine)-diethylene glycol ester (TosGLY_2_DEG) and di-*p*-toluenesulfonic acid bis-(glycine)-ethylene glycol ester (TosGLY_2_EG). Scheme 2 shows the reaction scheme between glycine and (1) DEG and (2) EG.

**Figure 1:**
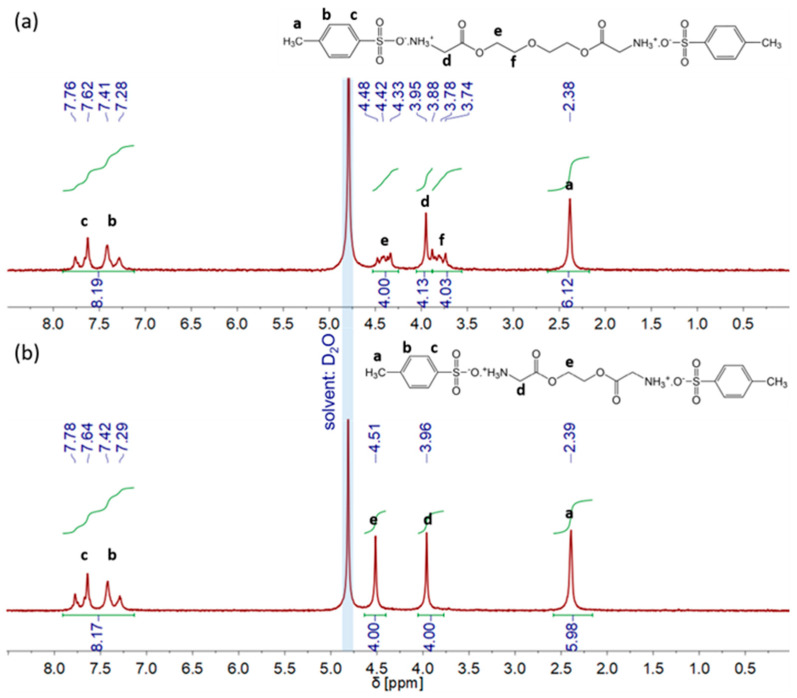
(**a**) ^1^H−NMR spectra of TosGLY_2_DEG and (**b**) TosGLY_2_EG.

^1^H-NMR spectra in Figure 1 show a successful synthesis of each glycine-based crosslinker in its tosylate form. The crosslinker was used without further purification for the next reaction step to form PASP-based hydrogels. In the subsequent crosslinking reaction, triethylamine (TEA) was added to form a salt with *p*-toluenesulfonic acid and thereby provide the reactive free amine groups of glycine for crosslinking of PSI chains.

#### 2.1.2. PSI Syntheses

**Figure 2:**
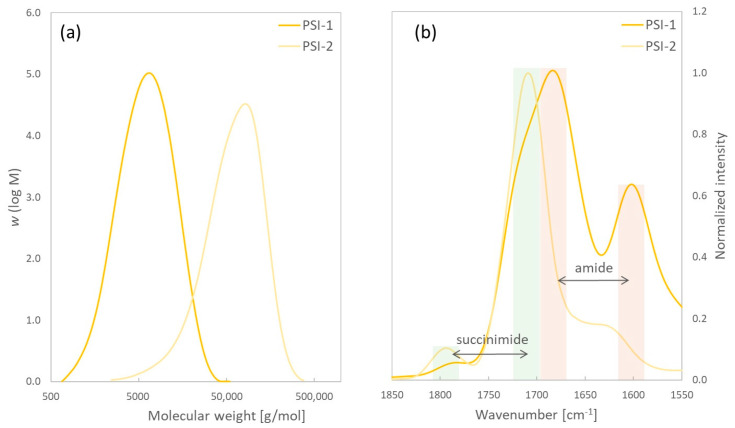
PSI−1 and PSI−2 characterization; (**a**) shows molar mass distribution of the polymers and (**b**) shows excerpt of FTIR spectra of PSI−1 and PSI−2 showing ν(C=O) region. Green bars highlight absorption corresponding to succinimide; red bars highlight amide groups.

PSI was successfully synthesized according to two different synthesis methods. PSI-1 was synthesized from maleic anhydride (MA) and urea in the presence of a mixture of sulfuric (VI) acid (H_2_SO_4_) and orthophosphoric (V) acid (H_3_PO_4_) [27]; PSI-2 was prepared in the presence of H_3_PO_4_ [28]. Both syntheses were carried out at 180 °C under reduced pressure. The yield of PSI samples from the two different syntheses was determined. Samples were characterized by GPC and FTIR for a structural analysis. Table 1 and Figure 2 show the results.

The molecular weight obtained from using MA and urea as monomers led to much lower values. The FTIR spectra of both synthesized PSI samples are shown in Figure 2b. The absorption bands at 1709 cm^−1^ and 1794 cm^−1^ correspond to ν(C=O) of succinimide. The appearance of both bands is explained by the two carbonyl groups in a close neighborhood in the PSI structure [29], which can be easily recognized in the PSI synthesized from ASP. The PSI sample synthesized from MA and urea shows these bands as a shoulder of the absorption at 1680 cm^−1^, which corresponds to the C=O-stretching vibration of an amide bond (amide I) together with the amide II peak at about 1600 cm^−1^. This indicates the presence of branched or ring-open(ed) structures. In a first step, aspartic acid is formed from the reaction between maleic anhydride/maleic acid and urea [30]. The main difference in the subsequent polymerization reaction compared to the procedure from ASP with H_3_PO_4_ is in this case the lower amount of acid during polycondensation: a ratio of acids to ASP is 1:2 (“ASP”) or 1:4 (“MA + urea”), respectively. This can cause ASP units to more preferably react with succinimide units undergoing aminolysis and ring-opening, leading to a branched structure. GPC data show that this reaction procedure also leads to shorter polymer chains. An advantage is generally a more homogeneous mixture due to the mixing of all reactants in the aqueous phase. The synthesis from ASP in combination with a high amount of phosphoric acid results in linear PSI chains with a high molecular weight as reported in study [28]. PSI synthesized from MA and urea was not able to form hydrogels after crosslinking. The short polymer chains and reduced reactive moieties due to a branched structure with less succinimide units prevent this material from being a feasible polymer to prepare PASP-based hydrogels. Therefore, PSI-2 was used in all hydrogel syntheses, and discussed hydrogels are based on this polymer.

### 2.2. Syntheses of Crosslinked PASP (clPASP) Hydrogels

Prepared PSI was crosslinked with diamine compounds such as HMD, the natural amino acid LYS, and the synthesized TosGLY_2_DEG and TosGLY_2_EG, which are converted to their free diamines during the crosslinking process. Crosslinking took place in a solution, while the exact procedure varied slightly depending on the used crosslinker system. In the case of 20% LYS as a crosslinker, additionally, Cu(II) and Zn(II) loading was carried out in situ. Crosslinked PSI (clPSI) was formed. Afterwards, clPSI was hydrolyzed with NaOH to form crosslinked poly(aspartic acid) gels in their sodium salt form (clPASP). The hydrogels were characterized using FTIR, regarding their swelling properties (kinetics, AUL) and exemplary SEM, and biodegradation tests were carried out. Table 2 summarizes the hydrogels that were prepared in this study.

#### 2.2.1. Spectroscopic Characterization of clPASP Hydrogels

**Figure 3:**
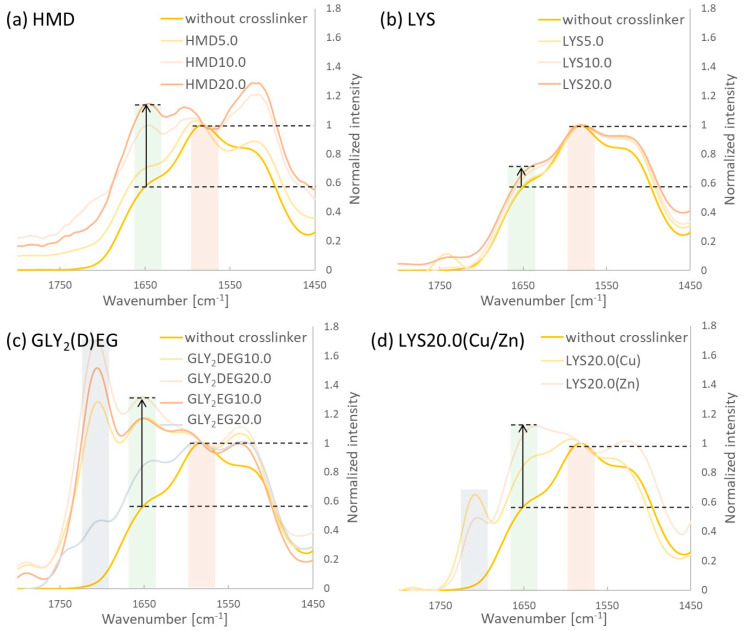
FTIR spectra of crosslinked PASP using different crosslinking agents and in situ crosslinking/loading with Cu(II)/Zn(II): (**a**) HMD; (**b**) LYS; (**c**) GLY_2_(D)EG; and (**d**) LYS20.0(Cu/Zn). Bars in the spectra indicate the C=O absorptions (red: carboxylate salt, green: amide, blue: ester (GLY_2_(D)EG) or carboxylic acid (LYS), respectively).

The FTIR spectra of linear PASP and the different crosslinked clPASP gels are shown in Figure 3. The disappearance of the absorption band at about 1710 cm^−1^ corresponding to the C=O-stretching vibration of the succinimide structure and appearance of the C=O-stretching vibration of the carboxylate salt (COO^−^Na^+^ units from hydrolyzed polymer) at about 1580 cm^−1^ alongside an observable shoulder at about 1650 cm^−1^ (amide) show successful hydrolysis to PASP. Furthermore, the increase in the amide bound at about 1650 cm^−1^ compared to the one of the carboxylate salt shows an increase in amide groups. This indicates an increasing crosslinker amount in the hydrogels [31]. Hydrolysis of PSI units leads to the presence of one amide group and one unit of carboxylic acid salt, while crosslinking leads to one amide present in the PASP chain and one amide forming the crosslink to another chain. Therefore, the increased absorption correlates with a higher crosslinker amount. The results show that in all cases, an increasing crosslinker amount added in the reaction process leads to an increased amount in the hydrogels. It seems that more HMD is incorporated compared to LYS as the FTIR results indicate. This was also observed in much faster hydrogel formation using HMD compared to LYS. Upon crosslinking with the GLY derivatives, additionally, the C=O absorption of the ester of the crosslinker molecules can be seen in the spectra.

#### 2.2.2. Swelling Properties of clPASP Hydrogels

The effect of the crosslinker type and amount on the swelling properties of the PASP hydrogels was determined. Swelling degrees were measured as time-dependent in distilled water and subsequently fitted according to the Voigt model [32]. AUL was measured by applying 0.1 and 0.3 psi while swelling in a self-3D-printed PLA tube.

**Figure 4:**
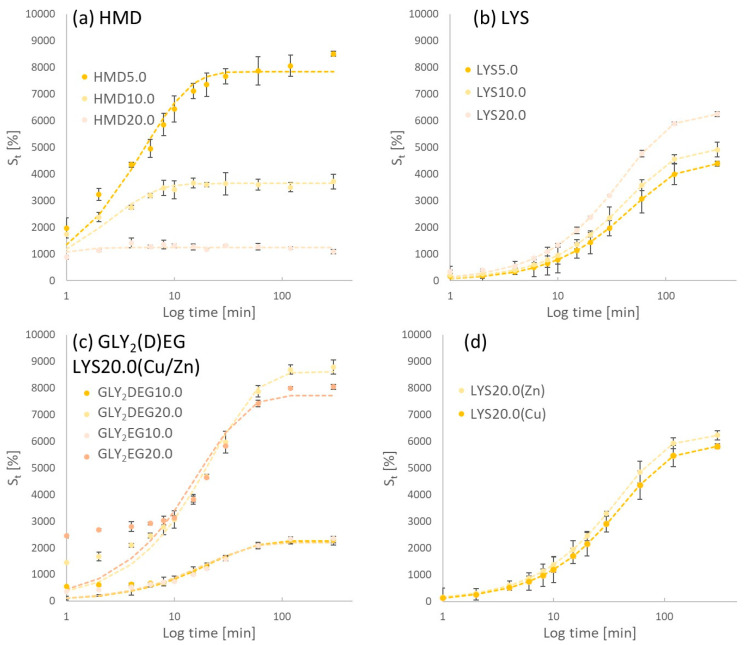
Swelling kinetics of (**a**) HMD, (**b**) LYS, (**c**) GLY_2_DEG and GLY_2_EG, and (**d**) LYS20.0(Cu) and LYS20.0(Zn) hydrogels.

Figure 4 shows the experimentally determined swelling degrees dependent on swelling time in distilled water of clPASP crosslinked with HMD, LYS, and GLY_2_(D)EG as well as the in situ-crosslinked and Cu/Zn-loaded hydrogels, respectively.

Depending on the crosslinker type and amount, generally, swelling degrees between 1000 and 9000% were reached. Hydrogels prepared with in situ Cu/Zn-loading both have a comparable swelling degree of about 5800% (LYS20.0(Cu)) and 6200% (LYS20.0(Zn)). In a comparison between HMD- and LYS-crosslinked gels, the ones with HMD obtained a higher swelling degree despite the presumed higher crosslinking (see Figure 4). It seems that LYS decreases water uptake compared to HMD, while an increase in LYS crosslinking and modification leads to an increase in water uptake. Via fitting regarding the Voigt model, the parameters S_max_ and t_63_ were determined as shown in Table 3.

Crosslinked hydrogels with HMD show a decrease in S_max_ with an increase in crosslinkers alongside a decrease in t_63_, showing a faster uptake of water, which is expected [33]. Nevertheless, gels crosslinked with LYS and GLY_2_(D)EG showed a higher S_max_ with an increase in crosslinkers. The value of t_63_ decreases slightly as observed for HMD-crosslinked hydrogels, which indicate a higher crosslinking density nevertheless. An explanation could be an incomplete crosslinking when using these crosslinkers. Leaving one of the two amino groups of the crosslinker pendant on the polymer chains leads to a derivatization of the hydrogels. This could contribute to a higher water uptake. Generally, crosslinker incorporation using GLY_2_(D)EG was less efficient compared to HMD and LYS, because no hydrogels were formed at all using a 5% crosslinker. Using LYS, crosslinking times were much higher compared to HMD. Both indicate less reactivity and therefore a derivatization alongside the crosslinking reaction, leaving pendant groups in the polymer network after hydrolysis. In the case of LYS, this provides an additional amine and carboxylic acid group. Their hydrophilicity could explain the increasing water uptake with an increasing addition of LYS while crosslinking.

**Figure 5:**
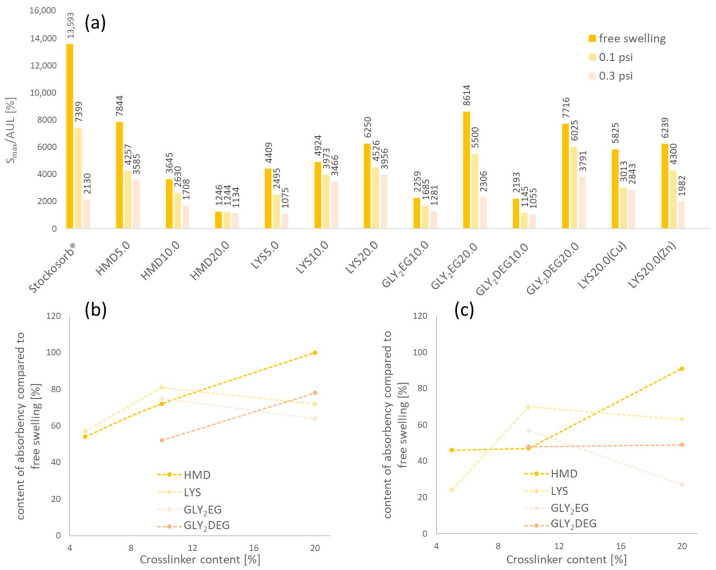
Absorbency under load (AUL), (**a**) measured for the different hydrogels (applied load was 0.1 or 0.3 psi, respectively); residual content of swelling dependent on crosslinker type and amount under (**b**) 0.1 psi and (**c**) 0.3 psi.

The swelling equilibrium under load S_AUL_ was measured after 2 h, applying 0.1 and 0.3 psi weight, respectively, resulting in the values S_(0.1 psi; 2 h)_ and S_(0.3 psi; 2 h)_. Figure 5 shows the results of the measurements. Additionally to the clPASP hydrogels, the polyacrylate-based hydrogel Stockosorb^®^ (Evonik, Essen, Germany) was tested in AUL measurements.

Under load, the water uptake decreases drastically, depending on the different hydrogel–crosslinker systems. However, this effect is generally less prominent in the case of PASP hydrogels with different crosslinkers and crosslinker content in comparison to a Stockosorb^®^ PAA hydrogel. Under 0.3 psi, various PASP hydrogels even had a higher swelling degree than the PAA gel. HMD-crosslinked hydrogels show a linear increase in residual swelling under load compared to free swelling with an increasing amount of HMD. Higher HMD contents lead to less reduction in water uptake under load compared to free swelling. Nevertheless, the absolute S_AUL_ is still higher with less crosslinkers. This relation is not strictly the case for the other prepared PASP hydrogels. This could be explained by the less reactive crosslinkers LYS and (D)EG derivatives compared to HMD. The crosslinking and functionalization of hydrogels happen in parallel and the hydrogel chemistry is changed additionally to a crosslinking of the polymer chains. This seems to affect AUL and free swelling. Generally, we can conclude that water uptake of the synthesized PASP-based hydrogels is less sensitive under load. The samples LYS10.0/20.0 and GLY_2_DEG20.0 especially show good swelling properties under load. A possibility to improve AUL of hydrogels is surface crosslinking of the gel particles. Surface crosslinking and a generally denser polymer network decrease S_max_ but lead to higher AUL [34]. Ashkani et al., synthesized hydrogels from different crosslinked polymers. A hydrogel based on poly(2-acrylamido-2-methyl-propane sulfonic acid) showed the best AUL compared to other gels based on PAA and polyacrylamide [35]. The presence of the methyl propane sulfonic acid groups in this polymer as bulky side groups is one structural difference between the studied hydrogels and could contribute to this effect. As discussed before, LYS or (D)EG derivatives could be less present as crosslinkers but also be incorporated as pendant groups in the gel network. Therefore, they act as bulky side groups as well, which could explain the improved AUL.

#### 2.2.3. Subsequent Loading with Micronutrients Cu and Zn

Hydrogels were loaded with the micronutrients copper or zinc, respectively. Two approaches were carried out. As already shown in Section 2.2.2, the two metal ions were included in the crosslinking process with LYS, which was used, on the one hand, as a crosslinker for PSI and, on the other hand, it should act as a chelating agent for Cu(II) and Zn(II). In this way, copper-loaded PSI was synthesized in one step during crosslinking. Subsequent hydrolysis led to the corresponding hydrogels. Swelling properties of such gels are already shown (Figure 4 and Figure 5 and Table 3). A second approach was the immersion of previously synthesized HMD-crosslinked hydrogels as well as linear PASP in the ion solutions of copper or zinc. Table 4 summarizes samples prepared in such a way.

**Figure 6:**
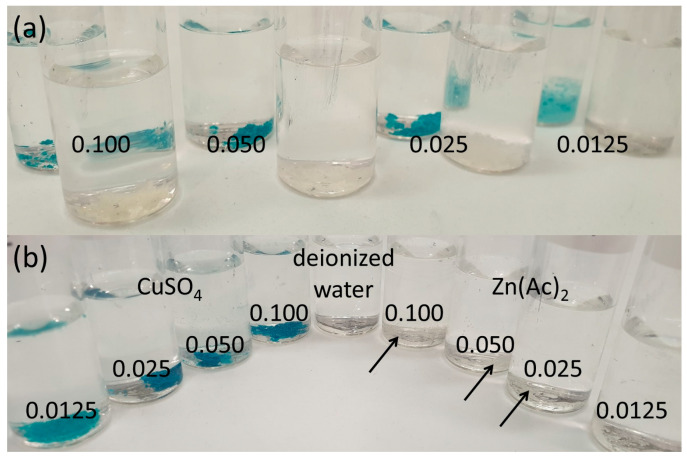
(**a**) HMD5.0 hydrogels loaded in Cu(II) and Zn(II) solutions with concentrations of 0.100, 0.050, 0.025, and 0.0125 mol/L, respectively, immersed in water and (**b**) linear PASP loaded in Cu(II) and Zn(II) solutions with concentrations of 0.100, 0.050, 0.025, and 0.0125 mol/L immersed in water, and an aqueous solution of linear PASP; arrows highlight visible precipitate.

Swelling of hydrogels in these solutions as well as swelling ratios of the resulting loaded hydrogels were measured. Figure 6 shows the HMD5.0 hydrogel and linear PASP mixtures with different concentrated Cu(II) or Zn(II) solutions.

**Figure 7:**
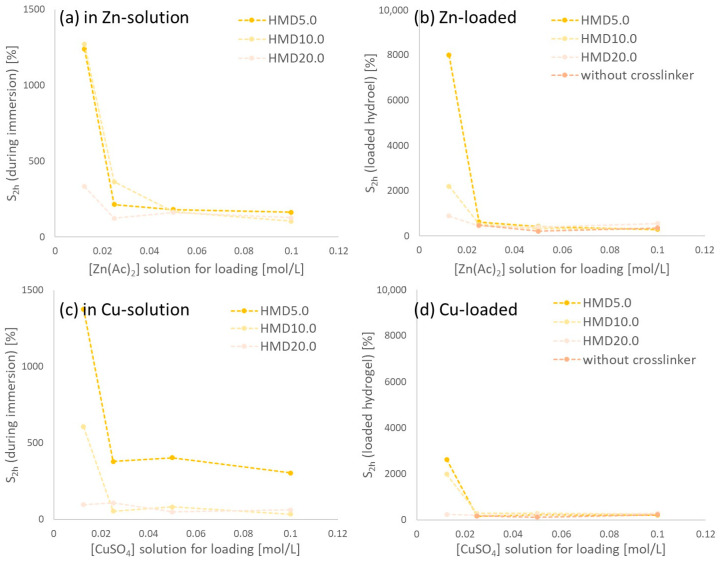
Swelling of hydrogels (**a**) during Zn-loading; (**b**) swelling of the corresponding loaded hydrogels in deionized water; (**c**,**d**) show the corresponding data for Cu-loading and loaded hydrogels.

Linear PASP immersed in metal salt solutions had a low water uptake with 477% being the highest value in this series (HMD5.0 in 0.025 mol/L Zn(II) solution) and was not considered further. Among the loaded PASP hydrogels, however, various samples maintained a considerable swelling degree. A higher concentration of metal ions leads to not or slightly swollen solids, while a decrease in metal ion concentration leads to swelling of the hydrogels in the solution. This can be explained by two reasons. Water uptake in higher-concentrated salt solutions is lower due to osmotic effects. Additionally, divalent ions such as Cu(II) and Zn(II) can contribute to a higher ionic crosslinking due to ionic interactions and complexation in PASP [36], which leads to less water uptake. Upon the addition of linear PASP to the ion solutions, a precipitate was observed compared to a clear solution of PASP in water. The insoluble polymer is explained by its dense ionic crosslinking due to high ion concentrations. PASP can act as a complexation agent for divalent ions such as Cu(II) or Zn(II) [36]. All loaded, washed, and dried polymers were immersed in deionized water to determine the swelling degrees after 2 h. Figure 7 shows the results of the swelling degrees of the gels. The linear PASP, precipitated in ion solutions shows no swelling. Loading HMD5.0 in a 0.0125 molar zinc solution leads to a hydrogel with a swelling degree of about 8000%, and the corresponding gel with copper reaches 2621%. Compared to other reported gels based on CMC or κ-carrageenan, a high water uptake was reached when loaded with zinc or copper ions [26,37]. Nevertheless, hydrogels based on carboxymethyl tamarind kernel gum loaded with zinc could be prepared with very high water uptake [38]. PASP, however, is a material with many possibilities of modification especially in its intermediate form PSI via aminolysis [15]. The incorporation of pendant groups to the network might not only tailor water uptake under different conditions, but could also introduce specific functionalities for chelating of (micro-)nutrient ions.

**Figure 8:**
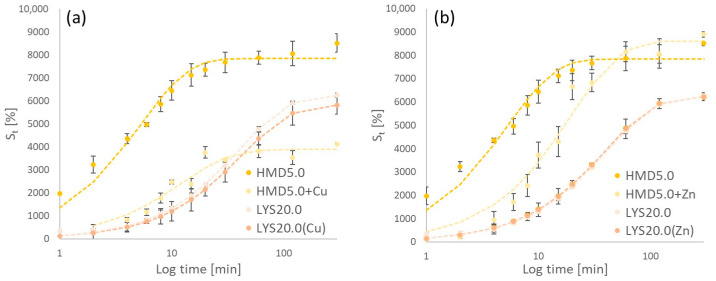
(**a**) Swelling kinetics of different Cu(II)-loaded hydrogels; (**b**) shows the data of the corresponding Zn(II)-loaded gels.

Figure 8 shows the swelling properties of the in situ-loaded LYS20.0(Cu/Zn) in comparison to the solely LYS-crosslinked counterpart as well as the HMD5.0 + Cu/Zn hydrogels with their unloaded counterpart in distilled water. Kinetic parameters are compared in Table 5.

In situ LYS-crosslinked and loaded hydrogels show similar swelling properties compared to the solely LYS-crosslinked gels. Loading with copper ions decreases the water uptake. S_max_ of subsequent loaded hydrogels differs more from the pure gel. In the case of Zn(II) loading, an increase is observed. Loading with copper (II) leads to a decrease in S_max_. This correlates with the data shown in Figure 7. Copper ions have a higher binding affinity to the PASP polymer via complexation [36].

**Figure 9:**
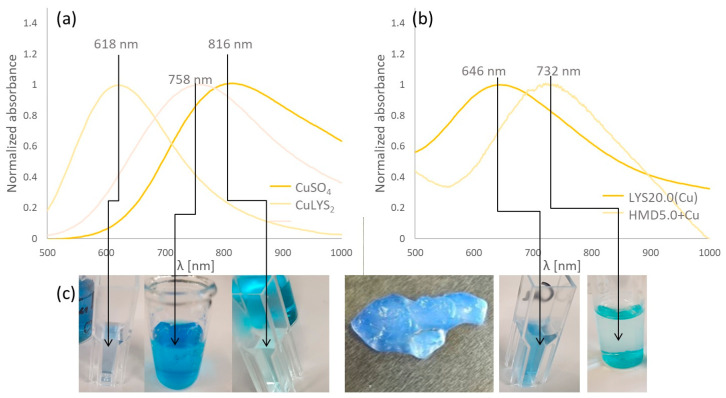
UV–VIS spectra of (**a**) different copper (II) species; (**b**) copper-loaded hydrogels LYS20.0(Cu) and HMD5.0 + Cu; (**c**) the corresponding solutions or hydrogels, from left to right: CuLYS_2_ and CuASP_2_ complexes, CuSO_4_ solution, a swollen LYS20.0(Cu) hydrogel sample, LYS20.0(Cu) and HMD5.0 + Cu hydrogels.

Additionally, UV-VIS spectra were recorded to monitor the copper species inside the hydrogel structure. Cu(II) could be present as precipitated or absorbed sulfate or as a chelate complex with the aspartic acid units, with LYS, or with both. Figure 9 shows the recorded spectra.

The results give no clear indication if copper is bound via complexation with LYS or ASP units. However, the spectra show that copper is not solely immobilized in the gel as dissolved or precipitated sulfate. A shift of the broad absorption of CuSO_4_ at 816 nm is observed in all samples. This indicates that copper salts are bound to the hydrogel in different ways, allowing for a broad release profile of micronutrients.

#### 2.2.4. Morphological Analysis

**Figure 10:**
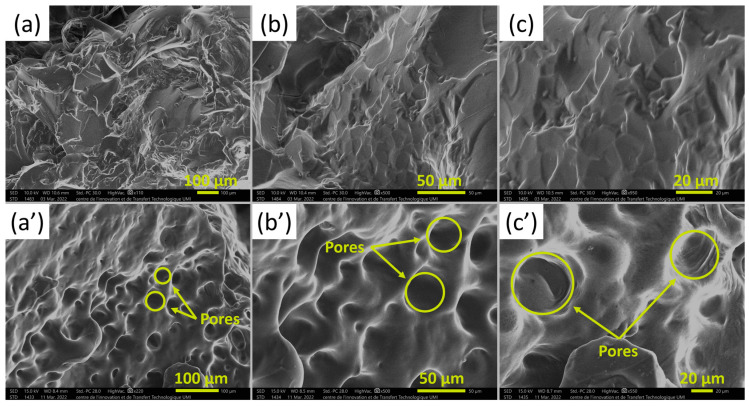
SEM micrographs of HMD5.0 (**a**–**c**) before and (**a’**–**c’**) after swelling in water and freeze drying in increasing magnification from left to right.

Figure 10 shows the SEM images taken exemplarily from an HMD5.0 hydrogel sample before and after swelling. SEM images of swollen and freeze-dried hydrogel show the presence of pores dispersed throughout the hydrogel. These pores were created when water entered the interior of the hydrogel, which ensure that HMD5.0 has excellent water absorption capacity [39,40].

#### 2.2.5. Biodegradation of Hydrogels

**Figure 11:**
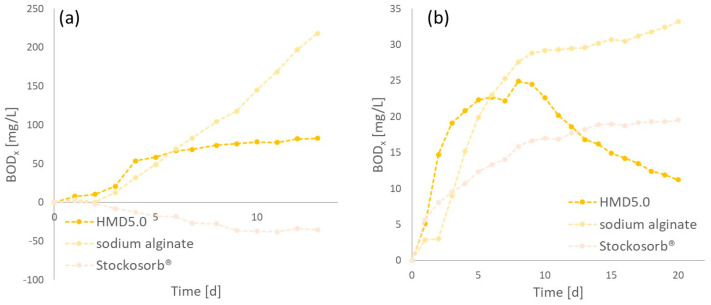
BOD−measurements of the polymers (HMD5.0, Stockosorb^®^, and sodium alginate) in (**a**) sewage sludge (return sludge from a municipal wastewater treatment plant in Jülich) and (**b**) in soil (taken from a garden in Jülich).

As an important feature of the hydrogels, biodegradability should be ensured. PASP and its crosslinked gels are reported to be biodegradable. In this study, HMD5.0 was exemplarily investigated regarding its biodegradability in sewage sludge and soil. In comparison, the PAA product Stockosorb^®^ and sodium alginate as controls were measured. Therefore, the biological oxygen demand (BOD) as an indicator for microbial activity was determined over time for the mentioned polymers in both systems (sewage sludge and soil). To determine the activity, the BOD of a control, e.g., sewage sludge, and soil without hydrogel was measured. Figure 11 shows the various measured BODs (vs. control) dependent on time.

A BOD below zero signifies less activity than the control, and a positive BOD signifies more degradation. Therefore, higher BOD values indicate higher biodegradation. In sewage sludge, all three hydrogels seem to show a lag phase of two days, until the degradation starts. This lag could be due to a change in salinity because of the swelling of the gels, leading to an increase in salt concentration. As shown above, alginate is very easily biodegradable, whereas crosslinked PASP (HMD5.0) has a lower biodegradability. Crosslinked PAA seems to be not biodegradable. Furthermore, the data indicate that PAA inhibits microbial activity in sludge since it has a lower BOD than sludge without hydrogels. Figure 11 also shows the degradation of hydrogels in soil. The graphs indicate that sodium alginate and HMD5.0 are better degradable than PAA. Nevertheless, the data show some degradability of PAA in soil. What is striking is the decline in BOD for HMD5.0 after 9 d. It signifies that other processes generate pressure/gas, other than carbon dioxide. This influences the test system. Nitrification and denitrification processes could cause the release of nitrogen and therefore a rising pressure in the flasks, which affects the measured values.

Since no inhibition chemical for nitrification was added, this could be the reason for the high BOD of PAA. In future experiments, this will be determined and biodegradation tests will be optimized for hydrogels.

## 3. Conclusions and Outlook

This paper presents novel PASP hydrogels for possible agricultural application as water and micronutrient reservoirs. The influence of different diamine crosslinkers on swelling properties, including AUL, was studied. Hydrogels were loaded with the micronutrients copper and zinc, respectively. Morphology and biological degradation in water and soil were analyzed exemplarily. PASP hydrogels were prepared from PSI via crosslinking with commercially available crosslinkers (HMD, LYS) and newly developed crosslinkers (GLY_2_(D)EG). Loading with copper (II) and zinc (II) was carried out in situ during crosslinking with LYS and before hydrolysis as well as via immersion of hydrolyzed polymers in corresponding metal salt solutions.

Crosslinking was successfully carried out in the range from 5 to 20% with HMD and LYS, while the incorporation of crosslinker GLY_2_(D)EG seems to be limited due to reduced nucleophilicity of α-amino groups. Higher amounts of crosslinkers are needed to form a stable hydrogel rather than water-soluble PASP. The same refers to in situ crosslinked and metal-loaded hydrogels. Crosslinked PSI was hydrolyzed to form the corresponding hydrogels. The swelling test of the hydrogels showed that the hydrogels prepared with EG- or DEG-coupled GLY had higher absorbencies with higher crosslinker amounts. The best water absorption capacity was marked for hydrogel crosslinked with GLY_2_DEG with an amount of 20% (S_max_ = 8614%). PASP hydrogel showed an improved biodegradability compared to a PAA-based product.

Further interest and work will be focused on the additional feature of the incorporation of nutrients—as already addressed using copper or zinc in a crosslinker system as micronutrients. The release of such and additional nutrient carriers will be part of future work as well as their usability in agriculture.

## 4. Materials and Methods

### 4.1. Materials

D,L-Aspartic acid (ASP), 1,6-hexamethylenediamine (HMD), maleic anhydride (MA), urea, orthophosphoric (V) acid (H_3_PO_4_, 85%), sulfuric (VI) acid (H_2_SO_4_, 98%), L-lysine (LYS), glycine (GLY), sodium hydroxide (NaOH), N,N′-dimethylformamide (DMF), dimethyl sulfoxide (DMSO), ethylene glycol (EG), diethylene glycol (DEG), triethylamine (TEA), anhydrous copper(II) sulfate (CuSO_4_), zinc acetate (ZnC₄H₆O₄), and sodium alginate (1% in H_2_O solution; viscosity: 15 cps; pH: 7.0) were purchased from Sigma-Aldrich, Burlington, MA, USA. As PAA-based hydrogel, the commercial product Stockosorb^®^ was purchased from GEFA Produkte^®^ Fabritz GmbH. The acrylic acid units were partially neutralized in the potassium salt form. Technical-grade toluene, ethyl acetate, isopropanol, methanol, and ethanol were used.

### 4.2. Syntheses

#### 4.2.1. Crosslinker Syntheses (GLY_2_(D)EG)

Di-*p*-toluenesulfonate of bis-(glycine)-diol ester (GLY_2_DEG or GLY_2_EG, respectively) crosslinker: The products were synthesized according to the following protocol. In a round-bottom flask, equipped with a Dean–Stark trap and condenser, glycine (0.028 mol), diol (DEG or EG) (0.13 mol), and *p*-toluenesulfonic acid monohydrate (0.030 mol) were dissolved in 60 mL of toluene by stirring. The reaction mixture was heated under reflux for 10 h until no more water was separated. The excess of toluene was evaporated under reduced pressure and the resulting products were dissolved in methanol. Unreacted glycine was filtered off and the crude product was recrystallized from an isopropanol/ethyl acetate mixture [41].

^1^H-NMR spectra (D_2_O, 60 MHz, δ [ppm]):

(1) 7.56–7.21 (d, 8H, 2Ar-H), 4.34 (t, 4H, COOCH_2_CH_2_), 3.88 (s, 4H, NH_3_^+^CH_2_), 3.75 (t, 4H, COOCH_2_CH_2_), and 2.31 (s, 6H, Ar-CH_3_).
(2) 7.61–7.22 (d, 8H, 2Ar-H), 4.44 (s, 4H, COOCH_2_), 3.89 (s, 4H, NH_3_^+^CH_2_), and 3.75 and 2.32 (s, 6H, Ar-CH_3_).

#### 4.2.2. Syntheses of PSI Polymers

Synthesis of PSI from MA and urea catalyzed with phosphoric and sulfuric acid mixture (PSI-1): MA (9.8 g, 0.100 mol) was first reacted in water at 80 °C for 40 min to obtain maleic acid. Afterwards, urea (4.2 g, 0.070 mol) and H_2_SO_4_/H_3_PO_4_ (0.91 g/0.84 g, 0.009 mol/0.009 mol) were added and the reaction was carried out in a vacuum at 180 °C for two hours. The product was dissolved in DMF (10 mL per 1.0 g) and precipitated in water (30 mL at room temperature) in order to remove acid and unreacted residues. PSI was washed with water until neutral, filtered, and dried at 50 °C in a convection oven for 12 h. FTIR (ATR) measurements and a GPC analysis (N,N-dimethylacetamide with 0.3% LiBr, PMMA calibration) were carried out.

Synthesis of PSI from ASP using phosphoric acid (PSI-2): ASP (10.0 g, 0.075 mol) was mixed with H_3_PO_4_ (4.30 g, 0.037 mol) at room temperature to form a homogeneous mixture. The mixture was placed in a flask connected to a vacuum pump after being heated to 180 °C. Pressure was about 20 mbar during 3 h of reaction time. Work-up and characterization were performed as described for PSI-1.

#### 4.2.3. Crosslinking of PSI Chains to Form Crosslinked PSI (clPSI)

Crosslinking with HMD: PSI (2.0 g, 0.021 mmol) was dissolved in DMF. HMD was added at room temperature as a 10 wt.% solution in ethanol. After approx. 15–25 min, an elastic gel was formed. After a total of 2.5 h, the gel was crushed, washed thoroughly with ethanol, and dried in a vacuum at 100 °C for 12 h in order to remove ethanol and any solvent residues of DMF. Table 6 summarizes reactant ratios and obtained product yield.

Crosslinking by LYS: PSI (2.0 g, 0.021 mol) was dissolved in 10 mL of DMSO. LYS was added at room temperature as a 10 wt.% solution in methanol. The solution was heated to 80 °C and stirred for an additional 24 h until an elastic gel was formed. Afterwards, the gel was crushed, washed thoroughly with ethanol, and dried in a vacuum at 100 °C for 12 h. Table 7 summarizes reactant ratios and obtained product yield.

Crosslinking by GLY_2_(D)EG: PSI (2.0 g, 0.021 mol) was dissolved in 10 mL of DMF. Bis(glycine)-diol (DEG or EG) was diluted with 4.0 mL of DMF before addition to the PSI solution. Afterwards, TEA was added dropwise at room temperature to form a homogeneous mixture. After a total reaction time of 24 h at 80 °C, an elastic gel was formed. The gel was crushed, washed thoroughly with ethanol, and dried in a vacuum at 100 °C for 12 h. Table 8 summarizes the reactant ratios and obtained product yield.

#### 4.2.4. Loading of Hydrogels with Micronutrients Cu/Zn

Subsequent loading of hydrogels with micronutrients Cu/Zn via swelling in ion solutions: An amount of 50 +/− 1 mg of the dry hydrogel sample and linear PASP was placed in 10.0 mL of 0.100, 0.050, 0.025, and 0.0125 mol/L solutions of CuSO_4_ or (Zn(Ac)_2_). After 2 h, the gels were filtered, washed, and dried at 50 °C. Swelling degrees were determined. From clPASP-HMD5.0 and clPASP-LYS20.0 loaded in the 0.0125 mol/L solution of zinc(II) and copper(II), respectively, swelling kinetics were measured as described below.

In situ loading of hydrogels with micronutrients Cu/Zn: PSI (1.0 g, 0.010 mol) was dissolved in 28.0 mL of DMF, and a solution of LYS (0.64 g, 0.004 mol, in 8.0 mL of water) was added. Afterwards, the mixture was stirred thoroughly at 60 °C for 1 h. TEA (629 μL, 0.004 mol) and CuSO_4_ (0.32 g, 0.021 mol) or Zn(Ac)_2_ (0.38 g, 0.021 mol), respectively, were mixed in 4.0 mL of water, diluted with 2.0 mL of DMSO, and afterwards added to the PSI solution. After addition, the mixture was stirred thoroughly at 80 °C for 3 h. The work-up was performed as described in Section 4.2.3.

#### 4.2.5. Hydrolysis of clPSI

To a 1.0 molar NaOH solution, clPSI samples were added while stirring vigorously. Directly, crosslinked polyaspartic acid (clPASP) was formed, which can be recognized by swelling of the solid and the rapid absorption of the NaOH solution. The mixture was reacted for 2 h before the product was washed with water (200 mL) and ethanol (40 mL) and dried at 50 °C. Table 9 summarizes the used amounts and ratios of clPSI and NaOH and obtained product yield of clPASP hydrogels.

### 4.3. Characterization Methods

^1^H NMR analysis: ^1^H-NMR spectra were measured with a 60 MHz spectrometer (Magritek Spinsolve 60) in D_2_O.

Gel permeation chromatography (GPC): GPC measurements were carried out using a GPC apparatus from Agilent. Samples were prepared and measured in dimethylacetamide containing 0.3% LiBr (DMAc/0.3LiBr). The GPC was equipped with one GRAM 30A (porosity: 100–10,000 Å) and two GRAM 1000A (porosity: 1000–1,000,000 Å) columns at 50 °C. The flow rate was set at 1.0 mL/min. The signal was recorded using an RI detector and calibration was performed with poly(methyl methacrylate) (PMMA).

FTIR spectroscopy: Spectra were measured with a Bruker TENSOR 27. The measurements were carried out as the ATR method of ground samples.

UV–VIS spectroscopy: Spectra were measured with a DR6000 spectrophotometer from Hach in an aqueous solution or via placing swollen gels in PMMA cuvettes.

Swelling properties: About 0.2 g of the superabsorbent was placed within two tea bags closed with a cable tie. The prepared sample was immersed in deionized water at 20 °C. The free swelling rate dependent on swelling time S_t_ was calculated using the following formula:

S_t_ = (m_s,t_ − m_d_)/m_d_∙100 [%], m_s,t_: swollen mass [g] at time t [min], m_d_: dry mass [g] (1)

Maximum swelling ratio S_max_ and t_63_ (time, after 63% of S_max_) were determined mathematically using the following equation (Voigt model) [32]:

S_t_ = S_max_∙(1 − e^−t/t63^) (2)

Absorbency under load (AUL) was measured using 3D-printed PLA tubes with an inner diameter of 2.5 cm and a height of 7.0 cm. The bottom of the tube was equipped with filter paper. About 0.16 g of hydrogel was weighed exactly and placed on the filter paper. A weight corresponding to 0.1 or 0.3 psi, respectively, was placed in the tube. The equipped tube was placed in a crystallizing dish with a diameter of 6 cm, which was filled with 60 mL of deionized water. After 2 h, the weight was determined. PLA tubes did not significantly take up humidity while being placed in the water and therefore water uptake of hydrogels could be determined by the subtraction of the sum of mass of the dry tube, filter paper, and weight from the mass after swelling with hydrogel.

Scanning electron microscopy (SEM): The surface morphology of superabsorbent polymer PASP before and after swelling was observed using a scanning electron microscope (SEM) (JSM-IT500HR). The swollen samples were freeze-dried prior to the SEM analysis.

Biodegradation of hydrogels: The degradation of PASP was determined in aquatic conditions and in soil. Stockosorb^®^ and sodium alginate were used as controls.

Aquatic conditions: Return sludge from a municipal wastewater treatment plant in Jülich was used. The dry mass content was at 3.0 g/L. A mixture of sewage sludge and tap water was utilized with a dry mass content of 0.4 g/L. Per 1.0 mL of the mixture, 5.0 mg of HMD5.0 and Stockosorb^®^ hydrogel was utilized. Sodium alginate concentration was 1.0 mg/mL. Two drops of allylthiourea were added to inhibit the nitrification reaction.

Soil: A soil sample was taken from a garden in Jülich. The sample was taken at a depth of about 10 cm to exclude most roots and was sieved with 4.0 mm mesh to remove bigger particles. In total, 15.0 g of the soil was eluated with 15.0 mL of deionized water. The eluate was used for hydrogel swelling. This resulted in a maximum amount of water per gram of hydrogel that can be taken up by the hydrogel. This amount was added to the soil to obtain a maximum swollen hydrogel and the best possible conditions for the degradation of the hydrogel. An amount of 5.0 mg of hydrogel (HMD5.0 and Stockosorb^®^) per gram of soil was applied. Sodium alginate concentration was 1.0 mg per gram of soil.

The degradation was determined by measuring the biological oxygen demand (BOD) of sludge and soil with hydrogels. BOD OxiTop systems from WTW were utilized. The incubation took place in a thermal cabinet at 20 °C +/− 1 °C. The BOD values are determined as follows:

BOD_hydrogel_ = BOD_sludge or soil+hydrogel_ − BOD_sludge or soil_ (3)

## Figures and Tables

**Table 1:** Data from the two different PSI syntheses.

Sample	Reaction Method/ Monomers	Acid(s)	Ratio (Monomer(s)/Acid(s)) [mol:mol]	Yield [g] (%)	M_n_ [g/mol] (PDI)	Reaction Time [min]
PSI-1	MA + urea	H_3_PO_4_ + H_2_SO_4_	4:1	7.92 (89)	4850 (1.63)	180
PSI-2	ASP	H_3_PO_4_	2:1	8.64 (95)	41,700 (1.75)	

**Table 2:** Prepared hydrogels from the different crosslinker types.

Sample Name *	Used Crosslinker	Amounts of Crosslinker [%]
clPASP-HMD5.0	1,6-hexamethylenediamine	5.0
clPASP-HMD10.0		10.0
clPASP-HMD20.0		20.0
clPASP-LYS5.0	L-lysine	5.0
clPASP-LYS10.0		10.0
clPASP-LYS20.0		20.0
clPASP-GLY_2_DEG10.0	bis-(glycine)-diethylene glycol ester	10.0
clPASP-GLY_2_DEG20.0		20.0
clPASP-GLY_2_EG10.0	bis-(glycine)-ethylene glycol ester	10.0
clPASP-GLY_2_EG20.0		20.0
clPASP-LYS20.0(Cu)	L-lysine	20.0
clPASP-LYS20.0(Zn)		20.0

* Hereinafter, names will be shortened, leaving out clPASP, e.g., “HMD5.0” instead of “clPASP-HMD5.0”.

**Table 3:** Determined parameters S_max_ and t_63_ dependent on crosslinker type and content of the synthesized hydrogels in comparison to the PAA product Stockosorb^®^.

Sample Name	S_max_	t_63_
HMD5.0	7844	5.27
HMD10.0	3645	2.56
HMD20.0	1246	0.50
LYS5.0	4409	50.44
LYS10.0	4924	46.19
LYS20.0	6250	41.64
GLY_2_DEG10.0	2259	22.00
GLY_2_DEG20.0	8614	22.80
GLY_2_EG10.0	2193	19.79
GLY_2_EG20.0	7716	17.28
LYS20.0(Cu)	5825	43.45
LYS20.0(Zn)	6239	39.97
Stockosorb^®^	19,282	6.47

**Table 4:** Samples loaded and treated with different concentrated Cu(II) or Zn(II) solutions.

Sample Name	c (Cu/Zn Solution) [mol/L]
HMD20.0/10.0/5.0 + Zn0.100	0.100
HMD20.0/10.0/5.0 + Cu0.100	
PASP + Zn0.100	
PASP + Cu0.100	
HMD20.0/10.0/5.0 + Zn0.050	0.050
HMD20.0/10.0/5.0 + Cu0.050	
PASP + Zn0.050	
PASP + Cu0.050	
HMD20.0/10.0/5.0 + Zn0.025	0.025
HMD20.0/10.0/5.0 + Cu0.025	
PASP + Zn0.025	
PASP + Cu0.025	
HMD20.0/10.0/5.0 + Zn0.0125	0.0125
HMD20.0/10.0/5.0 + Cu0.0125	
PASP + Zn0.0125	
PASP + Cu0.0125	

**Table 5:** Determined parameters S_max_ and t_63_ of Cu/Zn-loaded hydrogels in comparison to the corresponding pure gel (HMD5.0 and LYS20.0, respectively).

Sample Name	S_max_	t_63_
HMD5.0	7844	5.27
HMD5.0 + Cu	3891	12.59
HMD5.0 + Zn	8618	19.70
LYS20.0	6250	41.64
LYS20.0(Cu)	5825	43.45
LYS20.0(Zn)	6239	39.97

**Table 6:** Variation of crosslinker amount (HMD) for synthesis of crosslinked PSI (clPSI-HMD).

Sample Name	m (PSI) [g]	Crosslinker Content [mol-%]	m (HMD)/ 2.0 g of PSI [g]	Yield [g]
clPSI-HMD5.0	2.0	5.0	0.12	2.26
clPSI-HMD10.0		10.0	0.24	2.52
clPSI-HMD20.0		20.0	0.48	2.30

**Table 7:** Variation of crosslinker amount (LYS) for synthesis of crosslinked PSI (clPSI-LYS).

Sample Name	m (PSI) [g]	Crosslinker Content [%]	m (LYS)/ 2.0 g of PSI [g]	Yield [g]
clPSI-LYS5.0	2.0	5.0	0.15	0.92
clPSI-LYS10.0		10.0	0.30	2.50
clPSI-LYS20.0		20.0	0.60	2.40

**Table 8:** Variation of crosslinker amount (GLY_2_(D)EG) for synthesis of crosslinked PSI (clPSI-GLY_2_(D)EG).

Sample Name	m (PSI) [g]	Crosslinker Content [%] *	m (GLY_2_(D)EG)/2.0 g of PSI [g]	TEA [µL]
clPSI-GLY_2_DEG10.0	2.0	10.0	1.25	629
clPSI-GLY_2_DEG20.0		20.0	2.50	1238
clPSI-GLY_2_EG10.0		10.0	1.16	629
clPSI-GLY_2_EG20.0		20.0	2.33	1238

* With 5.0% crosslinker, no hydrogels were formed.

**Table 9:** Hydrolysis of clPSI to clPASP with NaOH.

Sample Name	Used Mass of clPSI [g]	V (1.0 mol/L NaOH) per 1.0 g of clPSI [mL]	Yield [g]
HMD5.0	2.0	10	2.34
HMD10.0			2.30
HMD20.0			1.93
LYS5.0			0.80
LYS10.0			1.97
LYS20.0			1.50
GLY_2_DEG10.0			1.70
GLY_2_DEG20.0			1.20
GLY_2_EG10.0			1.36
GLY_2_EG20.0			1.12
LYS20.0(Cu)	1.0		0.60
LYS20.0(Zn)			0.70

## Data Availability

Data contained within the article.
